# Joint Testing of Genotypic and Gene-Environment Interaction Identified Novel Association for *BMP4* with Non-Syndromic CL/P in an Asian Population Using Data from an International Cleft Consortium

**DOI:** 10.1371/journal.pone.0109038

**Published:** 2014-10-10

**Authors:** Qianqian Chen, Hong Wang, Holger Schwender, Tianxiao Zhang, Jacqueline B. Hetmanski, Yah-Huei Wu Chou, Xiaoqian Ye, Vincent Yeow, Samuel S. Chong, Bo Zhang, Ethylin Wang Jabs, Margaret M. Parker, Alan F. Scott, Terri H. Beaty

**Affiliations:** 1 Department of Epidemiology and Biostatistics, School of Public Health, Peking University, Beijing, China; Ministry of Health Key Laboratory of Reproductive Health, Beijing, China; 2 Mathematical Institute, Heinrich Heine University Duesseldorf, Duesseldorf, Germany; 3 Division of Biology and Biomedical Sciences, Washington University, St. Louis, Missouri, United States of America; 4 Department of Epidemiology, Johns Hopkins Bloomberg School of Public Health, Baltimore, Maryland, United States of America; 5 Department of Medical Research, Chang Gung Memorial Hospital, Taipei, Taiwan; 6 Department of Genetics and Genomic Sciences, Mount Sinai School of Medicine, New York City, New York, United States of America; 7 Department of Plastic Surgery, K K Women's and Children's Hospital, Singapore, Singapore; 8 Department of Pediatrics, National University of Singapore, Singapore, Singapore; 9 Department of Engineering, Xi'an JiaoTong University, Xi'an, China; 10 Department of Pediatrics, Johns Hopkins University School of Medicine, Baltimore, Maryland, United States of America; Department of Genetics and Genomic Sciences, Mount Sinai School of Medicine, New York City, New York, United States of America; 11 Department of Medicine, Johns Hopkins University School of Medicine, Baltimore, Maryland, United States of America; University of Colorado, United States of America

## Abstract

**Background:**

Non-syndromic cleft lip with or without cleft palate (NSCL/P) is a common disorder with complex etiology. The Bone Morphogenetic Protein 4 gene (*BMP4*) has been considered a prime candidate gene with evidence accumulated from animal experimental studies, human linkage studies, as well as candidate gene association studies. The aim of the current study is to test for linkage and association between *BMP4* and NSCL/P that could be missed in genome-wide association studies (GWAS) when genotypic (G) main effects alone were considered.

**Methodology/Principal Findings:**

We performed the analysis considering G and interactions with multiple maternal environmental exposures using additive conditional logistic regression models in 895 Asian and 681 European complete NSCL/P trios. Single nucleotide polymorphisms (SNPs) that passed the quality control criteria among 122 genotyped and 25 imputed single nucleotide variants in and around the gene were used in analysis. Selected maternal environmental exposures during 3 months prior to and through the first trimester of pregnancy included any personal tobacco smoking, any environmental tobacco smoke in home, work place or any nearby places, any alcohol consumption and any use of multivitamin supplements. A novel significant association held for *rs7156227* among Asian NSCL/P and non-syndromic cleft lip and palate (NSCLP) trios after Bonferroni correction which was not seen when G main effects alone were considered in either allelic or genotypic transmission disequilibrium tests. Odds ratios for carrying one copy of the minor allele without maternal exposure to any of the four environmental exposures were 0.58 (95%CI = 0.44, 0.75) and 0.54 (95%CI = 0.40, 0.73) for Asian NSCL/P and NSCLP trios, respectively. The Bonferroni *P* values corrected for the total number of 117 tested SNPs were 0.0051 (asymptotic *P* = 4.39*10^−5^) and 0.0065 (asymptotic *P* = 5.54*10^−5^), accordingly. In European trios, no significant association was seen for any SNPs after Bonferroni corrections for the total number of 120 tested SNPs.

**Conclusions/Significance:**

Our findings add evidence from GWAS to support the role of *BMP4* in susceptibility to NSCL/P originally identified in linkage and candidate gene association studies.

## Introduction

Non-syndromic cleft lip with or without cleft palate (NSCL/P) is one of the most common human congenital malformations [1]. Multiple genes and several common environmental risk factors have been implicated in the etiology. There is also suggestive evidence of gene-environment interaction (GxE) for common maternal exposures plus the possibility of biological interactions between different genes, although the latter remains tentative [2]–[5]. Understanding of the genetic components of this common and complex birth defect has been greatly improved through recent genome-wide association studies (GWAS) where an association between *IRF6* and NSCL/P, first shown in linkage and candidate gene studies, has been confirmed and associations with markers in a novel region on 8q24 in Europeans [6], [7] and two novel genes *ABCA4* and *MAFB* in Asian populations were successfully identified [4], [8]. However, most of the susceptibility genes identified in previous linkage and candidate gene association studies have not been replicated in GWAS and confirmation studies for GWAS findings have not always obtained supportive results [9]–[13]. Recent studies jointly considering genotypic (G) and GxE interaction showed potential to identify associations missed in conventional GWAS analysis considering G main effects alone [14]–[16]. This approach may clarify some of the inconsistencies among existing findings.

The Bone Morphogenetic Protein 4 gene (*BMP4*) on chromosome 14q22-q23 is one of the most promising candidate genes for NSCL/P, with evidence shown in animal experiments [17]–[20], genome-wide linkage studies [21], [22], a candidate gene sequencing study [23] and several candidate gene association studies [24]–[29], although there is inconsistency among candidate gene association studies and lack of support from published GWAS reports [6]–[8], [ 30].

The aim of the current study is to test for linkage and association with NSCL/P for markers in and around *BMP4* that could be missed in GWAS when genotypic (G) main effects alone were considered where risk was estimated for a combination of exposed and unexposed carriers using 895 Asian and 681 European complete case-parent trios originally ascertained through an international cleft consortium [8], [31]. In our joint analysis of G and interactions with exposure to any maternal environmental tobacco smoke (ETS) and any multivitamin supplements (VIT) under an additive conditional logistic regression model, significant novel associations with NSCL/P and non-syndromic cleft lip and palate (NSCLP) were shown in Asian and Chinese trios for *rs7156227* after Bonferroni correction. This significance held after trios with exposure to either any maternal tobacco smoking (SMK) or any alcohol consumption (ALCOHOL) were dropped. Our findings provide supportive evidence for linkage and association between the *BMP4* gene and risk of NSCL/P.

## Materials and Methods

### Ethics statement

Study protocols were reviewed and approved by the institutional review boards (IRB) or ethical committees at all participating institutions. Adult participants (including biological parents of all probands and probands old enough to give their own consent/assent) provided written informed consent for themselves. Parents or guardians of the minor participants provided written informed consent for the child's participation.

Specifically, the approval IRBs or ethical committees include IRBs of local ethical committee in Philippines, KK Women's and Children's Hospital in Singapore, National University of Singapore, Chang Gung Memorial Hospital in Taiwan, affiliated Hospital of Qingdao University in China, second affiliated Hospital of Shandong University in China, Peking University in China, Hospital of Stomatology, Wuhan University in China, West China School/Hospital of Stomatology, Sichuan University in China, Yonsei University in South Korea, University of Iowa in the US, the US National Institute of Environmental Health Sciences, University of Pittsburgh in the US, Johns Hopkins University in the US, Utah State University in the US; Regional scientific-ethical committee in Denmark, Norwegian Data Inspectorate, as well as the regional committee on research ethics for western Norway.

### Study design

The current analysis used a case-parent trio design where three matched “pseudo-controls” were generated for each observed case given the parents' genotypes and the Mendelian inheritance assumption.

### Case-parent trios

Samples for the current study were drawn from an international cleft consortium originally recruited from 13 (7 Asian and 6 European/US) sites with the goal of identifying genes influencing oral clefts in a case-parent trio GWAS. 895 trios of Asian ancestry were recruited mainly from countries/regions in Asia (Philippines, Singapore, Taiwan, Shandong, Wuhan, Chengdu and Korea) although there were a few trios of Asian ancestry who were ascertained from Norway (n = 3) and Maryland (n = 1). Trios of European origin (n = 681) were recruited mainly from European countries (Denmark and Norway) and the US (Iowa, Maryland, Pittsburg and Utah). There were also 3 trios who were of European ancestry and recruited from Singapore. The final determination for racial group was based on principal components analyses (PCA) for 33,078 randomly selected, independent and highly polymorphic single nucleotide polymorphisms (SNPs) on all parents from the 13 sites [8].

Clinical assessment was carried out to check for other birth defects and developmental delays to confirm the diagnosis of NSCL/P status of the cases. Population-based ascertainment was used in Norway and cases from other sites were mainly ascertained through surgical treatment centers.

### Maternal exposure assessment

Maternal exposures during the critical peri-conceptional period (3 months prior to and through the first trimester) of pregnancy were obtained from direct interviews of the mothers using similar structured interview questions at each site (the same questionnaires were used in 8 sites: Singapore, Taiwan, Shandong, Wuhan, Chengdu, Korea, Maryland and Utah).

Four maternal exposures were assessed as simple yes/no responses: any personal tobacco smoking (SMK), any environmental tobacco smoke in home, workplace or any other nearby places (ETS), any alcohol consumption (ALCOHOL) and any use of multivitamin supplements (VIT, not limited to folate). Exposure rates varied considerably between Asian and European trios (Table 1).

**Table 1:** Number of complete NSCL/P trios by race, recruitment site, and maternal exposure to tobacco smoking, environmental tobacco smoke, alcohol consumption and multivitamin supplements during the critical peri-conceptional period of pregnancy from an international cleft consortium.

Race & recruitment site	# Trios	SMK			ETS			ALCOHOL			VIT		
		n	%	*NA	n	%	NA	n	%	NA	n	%	NA
													
Asian													
Norway	3	0	0.0	0	1	33.3	0	1	33.3	0	1	50.0	1
Maryland	1	0	0.0	0	0	-	1	0	0.0	0	1	100.0	0
Philippines	94	4	4.3	0	0	-	94	5	5.3	0	29	30.9	0
Singapore	56	5	8.9	0	14	25.0	0	2	3.6	0	27	48.2	0
Taiwan	218	15	6.9	0	113	52.3	2	8	3.7	0	44	20.3	1
Shandong	183	1	0.5	0	65	35.9	2	0	0.0	1	11	6.4	10
Wuhan	175	0	0.0	0	3	1.7	1	0	0.0	3	11	7.4	26
Chengdu	106	1	0.9	0	92	88.5	2	3	2.8	0	2	1.9	3
Korea	59	0	0.0	0	12	23.1	7	0	0.0	8	2	3.4	0
Subtotal	895	26	2.9	0	300	38.2	109	19	2.2	12	128	15.0	41
European													
Denmark	21	7	33.3	0	0	-	21	6	28.6	0	12	57.1	0
Norway	279	128	45.9	0	43	15.4	0	148	53.0	0	87	44.8	85
Iowa	44	13	29.6	0	0	-	44	31	70.5	0	32	74.4	1
Maryland	86	23	27.4	2	6	22.2	59	25	29.8	2	69	85.2	5
Pittsburgh	96	22	22.9	0	0	-	96	40	42.1	1	78	82.1	1
Utah	152	12	7.9	0	14	9.3	1	20	13.2	0	89	58.6	0
Singapore	3	1	33.3	0	1	33.3	0	1	33.3	0	3	100.0	0
subtotal	681	206	30.3	2	64	13.9	221	271	40.0	3	370	62.8	92

*NA: information missing.

Because of the low exposure rates for maternal ALCOHOL (19/883*100% = 2.2%) and SMK (26/895*100% = 2.9%) (Table 1), as well as the high proportion of overlap between exposures to maternal SMK and ETS among Asian trios (21 of 22 mothers who smoked were also exposed to ETS, see Tables S1&S2), our linkage and association analyses for Asian trios were limited to those with information on maternal ETS (n = 786), VIT (n = 854) and both ETS and VIT(n = 746, Table 1 and Table S1). For European trios, we tested interactions with each or a combination of the four investigated maternal exposures. The numbers of complete NSCL/P trios with information on maternal SMK, ALCOHOL, ETS, and VIT are 679, 678, 460, and 589, respectively (Table1 & Table S3). The number of trios that have information on all four exposures is 374 (Table S4).

### DNA, genotyping and genotypic imputation

DNA for cases and their biological parents were obtained from a variety of types of biological specimens: whole blood, buccal brush/swab, saliva, mouthwash or dried blood spots.

122 single nucleotide variants (SNVs) in and around *BMP4* were genotyped using the Illumina Human610-Quad v.1_B Bead Chip at the Center for Inherited Disease Research at the Johns Hopkins University of the US [8]. Based on the genomic structure of NM_130850.2 or NM_ 001202.3 for *BMP4* (http://genome.ucsc.edu/) [25], these SNVs are located 3′ (n = 114), introns (intron_1 for *rs762642* and intron_3 for *rs2071047*) and 5′ (n = 6) of the gene, respectively.

In addition, 25 imputed SNVs located between the two genotyped SNPs (*rs7152946* and *rs1001161*) which are adjacent to *rs7156227* were also used in the current study. Imputation was carried out based on the 1000 genomes reference population using IMPUTE2 [32] by the GENEVA coordinating center [33].

### Statistical analysis

#### (1) SNP quality control assessment

The 122 genotyped and 25 imputed SNVs were all screened in Asian and European trios separately by Haploview (v4.2, http://www.broadinstitute.org/haploview/) using parents' data [34]. SNPs that passed the quality control (QC) criteria among all genotyped and imputed SNVs in and around the gene were valid for association and linkage assessment. The QC flags for each SNP are: Mendelian errors >5%, missing genotyping call rate>5%, minor allele frequency (MAF) <1%, and deviates from Hardy-Weinberg Equilibrium (HWE, *P*>0.05/122 = 0.00041 for genotyped and *P*>0.05/25 = 0.002 for imputed SNVs, respectively).

For Asian trios, the numbers of SNPs used in analyses were 117 (110 genotyped plus 7 imputed for those informative for ETS or both ETS and VIT), and 118 (111 genotyped plus 7 imputed for those informative for VIT). 120 (113 genotyped plus 7 imputed) SNPs were used in all analyses for European trios. Specifically, 10 and 3 genotyped SNVs were dropped due to MAF<1% in Asian and European parents, respectively; other genotyped SNVs were dropped due to linkage disequilibrium (LD, as measured by r^2^ = 1) with another SNP. All 18 imputed SNVs were dropped due to MAF<1%. The lowest genotyping call rates for Asian and European trios were 99.2% and 98.7%, respectively (data not shown).

#### (2) Test of linkage and association considering genotypic main effect alone

The genotypic TDT (gTDT) has higher power to test for linkage and association between an observed marker allele and an unobserved causal gene when the G main effect term alone is included in a conditional logistic regression model i.e. [LogitP(case) = β_g_G] [35]. Under an additive model, genotype G is coded as 0, 1 or 2 to represent the number of minor alleles carried by cases and pseudo-controls [36], [37]. The direction and magnitude of association is reported as an estimated odds ratio [(OR) = exp(β_g_)] for those carrying one copy of the minor allele (in comparison to non-carriers) ignoring interaction with any environmental exposure. The corresponding OR in comparison to non-carriers was estimated as exp(2β_g_) for individuals carrying two copies of the minor allele. A 1 degree of freedom (df) Wald test is used to test for statistical significance for G in this model.

Findings for all 895 Asian and 681 European complete NSCL/P trios using allelic TDT for genotyped markers were published in our previous GWAS report [8], where there was no significant association detected in or near *BMP4*. In the current analysis, the gTDT was performed among subsets of NSCL/P trios with data on selected maternal environmental factors.

The number of trios contributing to test statistics varied across SNPs and maternal environmental exposures because of differences in genotyping call rates, exposure rates as well as the informativeness of the markers.

#### (3) Test of linkage and association jointly considering G and GxE interaction

To capture evidence of linkage and association between *BMP4* and NSCL/P that may be missed by the allelic or genotypic TDT, the additive conditional logistic regression model for gTDT was extended to include additional GxE terms as Logit[P(case)] = β_g_G+∑[β_(g*e_i)_G*E_i_] to jointly consider the effects of a genetic marker and interaction(s) with maternal environmental exposure(s). In this model, E_i_ represents a dummy variable denoting status for selected maternal environmental exposure [35], [38]. Linkage and association signals can be captured by the likelihood ratio test (LRT) comparing a full model containing both G and GxE terms to a null model without containing any terms. The degrees of freedom for the LRT can be computed by the number of parameters in the larger model minus that in the smaller, nested model. When E_i_ is coded as 0 and 1 for the unexposed and exposed respectively, exp(β_g_) represents the OR for being a case carrying one copy of the minor allele (compared to non-carriers) for unexposed trios to any environmental factors considered in the GxE interaction terms. While exp[β_g_+∑β_(g*e_i)_] estimates the OR of being a case carrying one copy of the minor allele for trios with any or any combination of the maternal environmental exposures considered in the GxE interaction terms. A 1df Wald test can be used to test for statistical significance for each term in the model. Relevant ORs for carrying two copies of the minor allele can be estimated as exp(2β_g_) and exp[2(β_g_+∑β_(g*e_i)_)], respectively. The corresponding 95% confidence intervals (CIs) for these ORs can be estimated considering the associated standard errors.

Because maternal exposure status is the same between the observed case and all three pseudo-controls, tests for the independent effects of any maternal environmental exposure are not possible. Risk for exposed carriers can only be assessed by comparison to exposed non-carriers, while estimation of OR for exposed carriers by comparison to either unexposed carriers or unexposed non-carriers is not possible under a case-parent trio design.

In the Asian trios, interactions with maternal ETS and VIT (in addition to G) were first individually tested and then simultaneously considered using additive conditional logistic regression models. Two and 3 df LRTs were used to identify significant linkage and associations accordingly. ORs for being a case carrying one copy of the minor allele were estimated for trios with various exposure statuses for ETS and VIT (exposed to both ETS and VIT, ETS only, VIT only, neither ETS nor VIT). To avoid the potential influence of ALCOHOL and SMK exposures on these tests, analyses simultaneously considering interactions with ETS and VIT were repeated for the most significant SNP (*rs7156227*) using trios with no exposure to either ALCOHOL or SMK. These repeated analysis were performed among all Asian combined NSCL/P trios (n = 704), Chinese only NSCL/P trios (n = 609), Asian and Chinese non-syndromic cleft lip only (NSCLO) and NSCLP trios, as well as NSCL/P trios from the two largest groups (Taiwan and Shandong). For European trios, interactions with each of the four investigated maternal environmental exposures were all considered individually and simultaneously (a 5df LRT). ORs were estimated for the two largest groups of European trios: trios without exposure to any of the four environmental factors (n = 64) and trios had exposure to VIT only (n = 98).

Statistical analyses considering G and one GxE terms were performed using TRIO Package in R (version 3.0.0) [35], available at http://www.bioconductor.org. Analyses considering G and more than one GxE terms were carried out using SPSS software package version 20.0 (IBM SPSS Statistics).

Statistical power for relevant tests was estimated using QUANTO software with Bonferroni corrected significance level (α = 0.05/117 (for trios informative for ETS and combined ETS&VIT) and 0.05/118 (for trios informative for VIT) for Asian and α = 0.05/120 for European trios) [39] (http://hydra.usc.edu/gxe/). Other parameters required for power estimation were obtained either from the parents' genotypic data (number of trios, MAF and maternal environmental exposure rates) or from the fitted conditional logistic regression models (ORs for corresponding G and GxE terms).

## Results

### Test of linkage and association considering genotypic main effect alone

The tests of linkage and association using gTDT considering the G main effects alone were performed among trios with information on selected maternal environmental exposures. Nominal significance (*P*<0.05) was seen for 8 SNPs in Asian (Table 2 and Table S5) and another 2 SNPs among European trios (Tables S6 to S9). The most significant SNPs identified in Asian (*rs7156227*) and European (*rs11157980*) trios were in 3′ of *BMP4* with OR = 0.74 (95%CI = 0.62, 0.88) (Table 2) and 1.32 (95%CI = 1.07, 1.63) (Table S7), respectively, among Asian trios with information on ETS and European trios with information on VIT. The corresponding Bonferroni corrected *P* values were 0.083 (asymptotic *P* = 7.1*10^−4^) (Table 2) and 1 (asymptotic *P* = 1.08*10^−2^) (Table S7), respectively.

**Table 2:** Nominally significant associations for NSCL/P with SNPs in and around *BMP4* jointly considering G and interaction with ETS using conditional logistic regression models in 786 complete Asian trios with data on ETS.

SNP name	Position	All Trios informative for ETS			Trios without exposure to ETS		
		MAF (%)	*OR* (95%CI) _GxE	*P_*2df LRT (G+GxETS)	MAF (%)	*OR* (95%CI)	*P*
*rs7152946*	54051076	11.9	1.82 (1.18, 2.83)	7.48*10^−3^	10.8	0.65 (0.49, 0.87)	3.41*10^−3^
*rs7156227*	54055337	20.9	1.55 (1.08, 2.23)	1.78*10^−4^	20.0	0.61 (0.49, 0.78)	4.52*10^−5^
*rs1380131*	54072858	9.4	0.91 (0.54, 1.54)	1.98*10^−2^	10.1	0.73 (0.53, 0.99)	4.32*10^−2^
*rs10483623*	54090077	7.0	0.78 (0.44, 1.37)	8.88*10^−2^	6.8	1.46 (1.03, 2.08)	3.50*10^−2^
*rs17126895*	54248083	41.0	0.83 (0.62, 1.12)	1.32*10^−1^	41.0	1.21 (1.01, 1.45)	4.46*10^−2^
*rs8017615*	54259598	9.5	0.62 (0.37, 1.03)	7.69*10^−2^	9.8	1.39 (1.03, 1.88)	3.36*10^−2^
*rs10498466*	54391813	48.0	0.89 (0.66, 1.19)	4.16*10^−2^	47.7	1.24 (1.04, 1.49)	1.76*10^−2^
*rs1957860*	54429355	11.7	1.42 (0.90, 2.26)	6.37*10^−2^	11.7	0.72 (0.54, 0.95)	1.99*10^−2^
*rs8014363*	54431575	11.3	1.51 (0.94, 2.41)	6.86*10^−2^	11.4	0.71 (0.53, 0.96)	2.33*10^−2^

SNP name	Position	Trios had exposure to ETS			All trios informative for ETS (gTDT)		
		MAF (%)	*OR* (95%CI)	*P*	MAF (%)	*OR* (95%CI)	*P*
*rs7152946*	54051076	13.7	1.18 (0.85, 1.65)	3.15*10^−1^	11.9	0.84 (0.68, 1.04)	1.14*10^−1^
*rs7156227*	54055337	22.2	0.95 (0.72, 1.25)	7.26*10^−1^	20.9	0.74 (0.62, 0.88)	7.10*10^−4^
*rs1380131*	54072858	8.4	0.66 (0.43, 1.01)	5.68*10^−2^	9.4	0.70 (0.55, 0.90)	5.83*10^−3^
*rs10483623*	54090077	7.2	1.14 (0.73, 1.77)	5.74*10^−1^	7.0	1.33 (1.01, 1.75)	4.45*10^−2^
*rs17126895*	54248083	40.9	1.01 (0.80, 1.26)	9.54*10^−1^	41.0	1.12 (0.97, 1.30)	1.10*10^−1^
*rs8017615*	54259598	8.9	0.85 (0.56, 1.30)	4.59*10^−1^	9.5	1.18 (0.92, 1.50)	1.94*10^−1^
*rs10498466*	54391813	48.3	1.10 (0.87, 1.39)	4.06*10^−1^	48.0	1.19 (1.03, 1.37)	1.69*10^−2^
*rs1957860*	54429355	11.8	1.02 (0.71, 1.47)	9.26*10^−1^	11.7	0.82 (0.65, 1.02)	7.14*10^−2^
*rs8014363*	54431575	11.2	1.07 (0.74, 1.55)	7.08*10^−1^	11.3	0.83 (0.66, 1.05)	1.19*10^−1^

### Test of linkage and association jointly considering G and GxE interaction

#### (1) Asian trios

When G and interaction with ETS and VIT were considered respectively, 20 of 110 genotyped SNPs around *BMP4* showed nominal significance of linkage and association with NSCL/P (Table 2 and Table S5). Significance held for one SNP (*rs7156227*) after Bonferroni correction when G and interaction with ETS were considered and a Bonferroni corrected *P* = 0.021 (asymptotic *P* = 1.78*10^−4^) for the 2df LRT. The estimated OR for being a case carrying one copy of the minor allele (MAF = 0.20) at *rs7156227* without maternal exposure to ETS was 0.61 (95%CI = 0.49, 0.78) compared to unexposed non-carriers. The Bonferroni corrected *P* value from the Wald test was 0.0053 (asymptotic *P* = 4.52*10^−5^) (Table 2).

When G and interactions with ETS and VIT were considered simultaneously using an additive conditional logistic regression model, significant linkage and association with NSCL/P remained for *rs7156227* after Bonferroni correction, and another 14 SNPs also showed nominal significance. The Bonferroni corrected *P* value for the 3df LRT was 0.048 (asymptotic *P* = 4.13*10^−4^) for *rs7156227* when comparing the full model with the null. The OR associated with NSCL/P for *rs7156227* was 0.58 (95%CI = 0.45, 0.76) for Asian trios without maternal exposure to either ETS or VIT when compared to unexposed non-carriers with a corrected *P* = 0.0052 (asymptotic *P* = 4.47*10^−5^) (Table S10 and Table 3). For trios with other exposure statuses (exposed on both ETS and VIT, exposed on ETS only, and exposed on VIT only), no significance was observed for *rs7156227* where statistical power to detect linkage and association was limited (power was only 0.1%, 0.1% and 1.0%, respectively) when the Bonferroni corrected significance level (0.05/117) was used (Table 3).

**Table 3:** Linkage and associations with NSCL/P for *rs7156227* near *BMP4* jointly considering G and interactions with ETS and VIT using conditional logistic regression models in 745 complete Asian trios with data on both ETS and VIT.

Exposure		#trios	MAF (%)	*OR* (95%CI)	*P*	Power (%)
ETS	VIT					
Yes	Yes	24	27.1	1.12 (0.66, 1.92)	6.69*10^−1^	0.1
Yes	No	270	21.6	0.95 (0.72, 1.27)	7.37*10^−1^	0.1
No	Yes	*73	19.9	0.69 (0.42, 1.11)	1.27*10^−1^	1.0
No	No	378	20.1	0.58 (0.45, 0.76)	4.47*10^−5^	63.6

*73: genotyping for SNP *rs7156227* was failed for mother of one trio.

*P*_3df LRT = 4.13*10^−4^.

Significant linkage and association with NSCL/P and NSCLP held for *rs7156227* among Asian and Chinese only trios after trios with maternal exposure to ALCOHOL and SMK were dropped (Table 4). The Bonferroni corrected *P* value for the 3df LRT was 0.041 (asymptotic *P* = 3.5*10^−4^) for Chinese only NSCLP trios. ORs for carrying one copy of the minor allele and without maternal exposure to any of the four environmental factors were 0.58 (95%CI = 0.44, 0.75) and 0.57 (95%CI = 0.43, 0.75) for Asian and Chinese only NSCL/P trios, respectively. Relevant ORs for Asian and Chinese NSCLP trios were 0.54 (95%CI = 0.40, 0.73) and 0.52 (95%CI = 0.37, 0.71), respectively. The corresponding Bonferroni corrected *P* values ranged from 0.0051 to 0.0087 (asymptotic *P* values ranged from 4.39*10^−5^ to 7.42*10^−5^). No evidence of significant linkage or association was seen for *rs7156227* among all Asian or Chinese only NSCLO trios, or for NSCL/P trios originally ascertained from Taiwan and Shandong without exposure to any of the four environmental factors after Bonferroni correction. The corresponding statistical power was 0.9%, 0.4%, 4.8% and 28.0%, respectively (when the Bonferroni corrected significance level (0.05/117) was used) (Table 4).

**Table 4:** Linkage and associations with NSCLO, NSCLP and NSCL/P for *rs7156227* near *BMP4* jointly considering G and interactions with ETS and VIT in Asian trios after those exposed to SMK and ALCOHOL were dropped.

Race/Site	cleft type	Trios without exposure to any of the four factors					GxETS		*P_*3df LRT
		# trios	MAF (%)	*OR* (95%CI)	*P*	Power (%)	*OR* (95%CI)	*P*	
Asian	NSCLO	89	18.8	0.72 (0.41, 1.28)	2.63*10^−1^	0.9	1.71 (0.80, 3.65)	1.63*10^−1^	3.90*10^−1^
	NSCLP	282	20.5	0.54 (0.40, 0.73)	5.54*10^−5^	59.5	1.51 (0.96, 2.38)	7.32*10^−2^	4.76*10^−4^
	NSCL/P	371	20.1	0.58 (0.44, 0.75)	4.39*10^−5^	62.3	1.60 (1.09, 2.36)	1.64*10^−2^	4.56*10^−4^
Chinese	NSCLO	75	20.0	0.78 (0.43, 1.41)	4.03*10^−1^	0.4	1.43 (0.64, 3.22)	3.84*10^−1^	3.18*10^−1^
	NSCLP	245	20.8	0.52 (0.37, 0.71)	5.74*10^−5^	57.8	1.56 (0.96, 2.52)	7.04*10^−2^	3.50*10^−4^
	NSCL/P	320	20.6	0.57 (0.43, 0.75)	7.42*10^−5^	57.1	1.56 (1.03, 2.35)	3.43*10^−2^	7.93*10^−4^
Taiwan	NSCL/P	76	23.7	0.58 (0.35, 0.97)	3.88*10^−2^	4.8	1.45 (0.73, 2.86)	2.86*10^−1^	1.98*10^−1^
ShanDong	NSCL/P	101	20.3	0.43 (0.25, 0.73)	1.94*10^−3^	28.0	1.77 (0.80, 3.91)	1.56*10^−1^	1.14*10^−2^

Race/Site	cleft type	All trios informative for ETS&VIT (gTDT)					GxVIT	
		# trios	MAF (%)	*OR* (95%CI)	*P*	Power (%)	*OR* (95%CI)	*P*
Asian	NSCLO	190	19.7	0.90 (0.62, 1.30)	5.71*10^−1^	0.2	0.70 (0.23, 2.10)	5.20*10^−1^
	NSCLP	513	21.3	0.67 (0.54, 0.84)	3.25*10^−4^	48.5	1.56 (0.85, 2.87)	1.54*10^−1^
	NSCL/P	704	20.9	0.72 (0.60, 0.87)	6.56*10^−4^	43.7	1.29 (0.76, 2.19)	3.54*10^−1^
Chinese	NSCLO	161	20.7	0.83 (0.56, 1.24)	3.66*10^−1^	0.5	0.46 (0.13, 1.61)	2.26*10^−1^
	NSCLP	448	21.3	0.67 (0.53, 0.85)	8.11*10^−4^	39.5	2.21 (1.04, 4.67)	3.82*10^−2^
	NSCL/P	609	21.1	0.71 (0.58, 0.87)	7.94*10^−4^	39.9	1.43 (0.77, 2.67)	2.61*10^−1^
Taiwan	NSCL/P	196	23.2	0.75 (0.53, 1.05)	8.98*10^−2^	2.9	1.48 (0.61, 3.60)	3.88*10^−1^
ShanDong	NSCL/P	170	21.3	0.57 (0.39, 0.84)	4.00*10^−3^	21.6	1.65 (0.46, 5.88)	4.39*10^−1^

Using imputed data, one SNP (*rs6572915*) 898 bp upstream of *rs7156227* also showed significant association with NSCL/P and NSCLP after Bonferroni correction. However, *rs6572915* is almost in complete LD (r^2^ = 0.996) with *rs7156227*.

#### (2) European trios

In the analysis of European trios considering G and interaction with four maternal environmental exposures individually using conditional logistic regression, 19 SNPs showed nominally significant associations with NSCL/P. The OR for carrying one copy of the minor allele and without exposure to VIT was 0.42 (95%CI = 0.23, 0.79) (asymptotic *P* = 7.18*10^−3^, Bonferroni corrected *P* = 0.86) for the most significant SNP (*rs210359*) (Table S7).

When interactions with all four maternal exposures were considered simultaneously in a single model, no association was seen for *rs7156227* (MAF = 28.9%, OR 1.34 (95%CI = 0.86, 2.06), asymptotic *P* = 0.19) among trios without exposure to any of the four exposures. Two other SNPs (*rs210327* and *rs1380131*) showed nominal significance in the 5df LRT, and another SNP (*rs210361*) showed nominal significant evidence among 64 trios without maternal exposure to any of the four environmental exposures. The 5df LRT gave *P* = 0.019 for the most significant SNP (*rs1380131*), but this was not significant after Bonferroni correction (Table S11). No SNP showed any significant association with NSCL/P among 98 trios exposed to VIT only when compared to exposed non-carriers after Bonferroni correction (data not shown).

No nominally significant association was seen for any of the 7 imputed SNPs around *rs7156227* in joint analysis of G and interactions with all four environmental exposures in one model for European trios (data not shown).

## Discussion

*BMP4* is among the genes known to be functionally involved in craniofacial development. Building upon its biological function, as well as evidence from animal experimental studies [17]–[20] and human linkage studies [21], [22], *BMP4* has been considered a leading candidate gene for NSCL/P and intensely studied in association studies using case-control [24], [28], [29] and case-parent trio designs [25]–[27]. Four of the 9 published candidate gene studies provided supportive evidence for association between *BMP4* and NSCL/P, where the identified SNPs included *rs17563* [24], [28], [29], *rs10130587* [25], *rs762642* [26] and *rs2855530* [27].

In the current study, we tested linkage and association using a strategy that jointly considers G and GxE interaction in an additive conditional logistic regression model. Using this approach, we identified a novel significant association with NSCL/P (and NSCLP) for SNP *rs7156227* near *BMP4* among Asian trios if the mother reported not having been exposed to any of the four environmental factors including ETS, VIT, ALCOHOL and SMK during the critical peri-conceptional period of pregnancy. This significant association was not seen in a previous GWAS search considering genetic main effects alone when genotypic risks were estimated for the combined exposed and unexposed carriers [8]. Our finding adds evidence from GWAS to support the role of *BMP4* in susceptibility to NSCL/P originally identified in candidate gene association studies. This is also the first report of association between a marker near *BMP4* and the NSCLP subgroup. As with the majority of findings from genetic association studies, the most significantly associated common SNP, *rs7156227*, is located in the 3′ noncoding region of *BMP4*. Although different SNPs have been previously associated with NSCL/P, linkage disequilibrium could account for this signal with some nearby unknown causal mutation(s). As with some previously published studies, there was no significant linkage and association identified in European trios after Bonferroni correction [25], [40] which may indicate insufficient power to detect association and/or etiological heterogeneity between Asian and European populations.

Considering the public health intervention potential, attention has been given to modifiable environmental risk factors, especially SMK, and ETS which may well interact with susceptibility genes for complex diseases [5], [41], [42]. In the current study, there was no evidence of linkage and association identified for gene-environment interaction between *BMP4* and NSCL/P either among all Asian or European trios when exposed carriers to selected environmental exposures were compared to corresponding exposed non-carriers under an additive conditional logistic regression model.

Because the analytical approach adopted in the current study allows separate estimation of risks for exposed and unexposed carriers, respectively, genetic and GxE interaction effects are potential to be identified that could be missed in analysis that considers genotypic main effects alone when risks for exposed and unexposed carriers are in opposite directions, or exist only in one of the exposure groups.

Also, because these analyses were performed in smaller groups compared to those with genotypic main effects considered alone, statistical power became a more important issue, especially when the Bonferroni corrected significance level was considered. For example, statistical power to test for linkage and association can be as low as 0.9% and 0.4% for Asian and Chinese only NSCLO trios without maternal exposure to any of the four factors. The statistical power was even lower for some other exposure groups due to smaller size and lower exposure rates. Larger numbers of case-parent trios will be needed to better answer questions about the effect of genotypic, especially gene-environment interactions in controlling risk of NSCL/P. In addition, because the case-parent trios design is naturally matched for parental exposures, the independent effects of maternal environmental exposures, as well as the interaction effects for exposed carriers in comparison to unexposed carriers or unexposed non-carriers, cannot be estimated. Other study designs, such as case control, may be better suited to answer specific questions about environmental factors alone [43] and gene-environment interaction [44].

Our study showed analyses jointly considering G and multiple GxE interactions can identify important genes through linkage and association tests that analyses considering G effects alone would miss. This analytical approach has provided supportive evidence from GWAS for *BMP4* as an important gene for NSCL/P and NSCLP in Asian trios.

## Supporting Information

Table S1 Maternal exposure to tobacco smoking, environmental tobacco smoke, multivitamin supplements and alcohol consumption in NSCL/P probands from 895 complete Asian trios.
(DOC)

Table S2 Combined maternal exposure to environmental tobacco smoke, multivitamin supplements, alcohol consumption and tobacco smoking in NSCL/P probands from 895 complete Asian trios.
(DOC)

Table S3 Maternal exposure to tobacco smoking, environmental tobacco smoke, multivitamin supplements and alcohol consumption in NSCL/P probands from 681 complete European trios.
(DOC)

Table S4 Combined maternal exposure to environmental tobacco smoke, multivitamin supplements, alcohol consumption and tobacco smoking in NSCL/P probands from 681 complete European trios.
(DOC)

Table S5 Nominally significant associations for NSCL/P with SNPs in and around *BMP4* jointly considering G and interaction with maternal VIT using conditional logistic regression models in 854 complete Asian trios informative for VIT.
(DOC)

Table S6 Nominally significant associations for NSCL/P with SNPs in and around *BMP4* jointly considering G and interaction with maternal ETS using conditional logistic regression models in 460 complete European trios informative for ETS.
(DOC)

Table S7 Nominally significant associations for NSCL/P with SNPs in and around *BMP4* jointly considering G and interaction with maternal VIT using conditional logistic regression models in 589 complete European trios informative for VIT.
(DOC)

Table S8 Significant and marginally significant associations for NSCL/P with SNPs in and around *BMP4* jointly considering G and interaction with maternal ALCOHOL using conditional logistic regression models in 678 complete European trios informative for ALCOHOL.
(DOC)

Table S9 Significant and marginally significant associations for NSCL/P with SNPs in and around *BMP4* jointly considering G and interaction with maternal SMK using conditional logistic regression models in 679 complete European trios informative for SMK.
(DOC)

Table S10 Nominally significant associations with NSCL/P for SNPs in and around *BMP4* jointly considering G and interactions with maternal ETS and VIT using conditional logistic regression models in 746 complete Asian trios informative for ETS and VIT.
(DOC)

Table S11 Nominally significant associations for NSCL/P with SNPs in and around *BMP4* jointly considering G and interactions with maternal SMK, ETS, ALCOHOL and VIT using conditional logistic regression models in 374 complete European trios informative for all four exposures.
(DOC)
