# Supplementary material for: Joint Testing of Genotypic and Gene-Environment Interaction Identified Novel Association for BMP4 with Non-Syndromic CL/P in an Asian Population Using Data from an International Cleft Consortium
Source: PLoS One. 2014 Oct 10;9(10):e109038. doi: 10.1371/journal.pone.0109038 (PMC4193821; doi:10.1371/journal.pone.0109038)
Supplement: Table S1 — Maternal exposure to tobacco smoking, environmental tobacco smoke, multivitamin supplements and alcohol consumption in NSCL/P probands from 895 complete Asian trios. (DOC) [file pone.0109038.s001.doc]

| Table S1 Maternal exposure to tobacco smoking, environmental tobacco smoke, multivitamin supplements and alcohol consumption in NSCL/P probands from 895 complete Asian trios | | | | | | | | | | | |
| --- | --- | --- | --- | --- | --- | --- | --- | --- | --- | --- | --- |
|  |  |  |  |  |  |  |  |  |  |  |  |
|  |  |  |  |  |  |  |  |  |  |  |  |
| Exposure | |  | ETS | | | | |  | *NA | Total | |
|  |  | Yes | |  | No | | Subtotal |  |  |  |  |
|  |  | n | % |  | n | % |  |  |  |  |  |
| SMK | Yes | 21 | 95.5 |  | 1 | 4.5 | 22 |  | 4 | 26 | |
|  | No | 279 | 36.5 |  | 485 | 63.5 | 764 |  | 105 | 869 | |
|  | Subtotal | 300 |  |  | 486 |  | 786 |  | 109 | 895 | |
|  | NA | 0 |  |  | 0 |  | 0 |  | 0 | 0 | |
|  |  |  |  |  |  |  |  |  |  |  | |
| ALCOHOL | Yes | 12 | 85.7 |  | 2 | 14.3 | 14 |  | 5 | 19 | |
|  | No | 288 | 37.7 |  | 475 | 62.3 | 763 |  | 101 | 864 | |
|  | Subtotal | 300 |  |  | 477 |  | 777 |  | 106 | 883 | |
|  | NA | 0 | 0.0 |  | 9 | 100.0 | 9 |  | 3 | 12 | |
|  |  |  |  |  |  |  |  |  |  |  | |
| VIT | Yes | 24 | 24.5 |  | 74 | 75.5 | 98 |  | 30 | 128 | |
|  | No | 270 | 41.7 |  | 378 | 58.3 | 648 |  | 78 | 726 | |
|  | Subtotal | 294 |  |  | 452 |  | 746 |  | 108 | 854 | |
|  | NA | 6 |  |  | 34 | 85.0 | 40 |  | 1 | 41 | |
|  |  |  |  |  |  |  |  |  |  |  | |
| Total |  | 300 |  |  | 486 |  | 786 |  | 109 | 895 | |
| *NA: information missing | | | | | | | | | | |  |
