# Supplementary material for: Joint Testing of Genotypic and Gene-Environment Interaction Identified Novel Association for BMP4 with Non-Syndromic CL/P in an Asian Population Using Data from an International Cleft Consortium
Source: PLoS One. 2014 Oct 10;9(10):e109038. doi: 10.1371/journal.pone.0109038 (PMC4193821; doi:10.1371/journal.pone.0109038)
Supplement: Table S2 — Combined maternal exposure to environmental tobacco smoke, multivitamin supplements, alcohol consumption and tobacco smoking in NSCL/P probands from 895 complete Asian trios. (DOC) [file pone.0109038.s002.doc]

| Table S2 Combined maternal exposure to environmental tobacco smoke, multivitamin supplements, alcohol consumption and tobacco smoking in NSCL/P probands from 895 complete Asian trios | | | | | | |
| --- | --- | --- | --- | --- | --- | --- |
|  |  |  |  |  |  |  |
|  |  |  |  |  |  |  |
|  |  |  |  |  |  |  |
| Exposure | Alcohol | SMK | ETS | | | Total |
|  |  |  | Yes | No | *NA |  |
| VIT Yes | Yes | Yes | 2 | 0 | 2 | 4 |
|  |  | No | 1 | 0 | 2 | 3 |
|  |  | NA | 0 | 0 | 0 | 0 |
|  | No | Yes | 3 | 0 | 1 | 4 |
|  |  | No | 18 | 74 | 25 | 117 |
|  |  | NA | 0 | 0 | 0 | 0 |
|  | NA | Yes | 0 | 0 | 0 | 0 |
|  |  | No | 0 | 0 | 0 | 0 |
|  |  | NA | 0 | 0 | 0 | 0 |
| VIT No | Yes | Yes | 5 | 1 | 1 | 7 |
|  |  | No | 4 | 0 | 0 | 4 |
|  |  | NA | 0 | 0 | 0 | 0 |
|  | No | Yes | 11 | 0 | 0 | 11 |
|  |  | No | 250 | 368 | 74 | 692 |
|  |  | NA | 0 | 0 | 0 | 0 |
|  | NA | Yes | 0 | 0 | 0 | 0 |
|  |  | No | 0 | 9 | 3 | 12 |
|  |  | NA | 0 | 0 | 0 | 0 |
| VIT NA | Yes | Yes | 0 | 0 | 0 | 0 |
|  |  | No | 0 | 1 | 0 | 1 |
|  |  | NA | 0 | 0 | 0 | 0 |
|  | No | Yes | 0 | 0 | 0 | 0 |
|  |  | No | 6 | 33 | 1 | 40 |
|  |  | NA | 0 | 0 | 0 | 0 |
|  | NA | Yes | 0 | 0 | 0 | 0 |
|  |  | No | 0 | 0 | 0 | 0 |
|  |  | NA | 0 | 0 | 0 | 0 |
| Total | | | 300 | 486 | 109 | 895 |
| *NA: information missing | | | | | | |
