# Supplementary material for: Joint Testing of Genotypic and Gene-Environment Interaction Identified Novel Association for BMP4 with Non-Syndromic CL/P in an Asian Population Using Data from an International Cleft Consortium
Source: PLoS One. 2014 Oct 10;9(10):e109038. doi: 10.1371/journal.pone.0109038 (PMC4193821; doi:10.1371/journal.pone.0109038)
Supplement: Table S3 — Maternal exposure to tobacco smoking, environmental tobacco smoke, multivitamin supplements and alcohol consumption in NSCL/P probands from 681 complete European trios. (DOC) [file pone.0109038.s003.doc]

| Table S3 Maternal exposure to tobacco smoking, environmental tobacco smoke, multivitamin supplements and alcohol consumption in NSCL/P probands from 681 complete European trios | | | | | | | | | | |  |
| --- | --- | --- | --- | --- | --- | --- | --- | --- | --- | --- | --- |
| Exposure | |  | | ETS | | | | | *NA | Total | |
|  |  | Yes | | |  | No | | Total |  |  |  |
|  |  | n | % | |  | n | % |  |  |  |  |
| SMK | Yes | 12 | 8.16 | |  | 135 | 91.84 | 147 | 59 | 206 | |
|  | No | 52 | 16.61 | |  | 261 | 83.39 | 313 | 160 | 473 | |
|  | subtotal | 64 | 13.91 | |  | 396 | 86.09 | 460 | 219 | 679 | |
|  | NA | 0 | - | |  | 0 |  | 0 | 2 | 2 | |
|  |  |  |  | |  |  |  |  |  |  | |
| ALCOHOL | Yes | 30 | 16.13 | |  | 156 | 83.87 | 186 | 85 | 271 | |
|  | No | 34 | 12.41 | |  | 240 | 87.59 | 274 | 133 | 407 | |
|  | subtotal | 64 | 13.91 | |  | 396 | 86.09 | 460 | 218 | 678 | |
|  | NA | 0 | - | |  | 0 |  | 0 | 3 | 3 | |
|  |  |  |  | |  |  |  |  |  |  | |
| VIT | Yes | 26 | 12.94 | |  | 175 | 87.06 | 201 | 169 | 370 | |
|  | No | 23 | 13.29 | |  | 150 | 86.71 | 173 | 46 | 219 | |
|  | subtotal | 49 | 13.10 | |  | 325 | 86.90 | 374 | 215 | 589 | |
|  | NA | 15 | 17.44 | |  | 71 | 82.56 | 86 | 6 | 92 | |
| Total |  | 64 |  | |  | 396 |  | 460 | 221 | 681 | |
| *NA: information missing | | | | | | | | | | |  |
