# Supplementary material for: Joint Testing of Genotypic and Gene-Environment Interaction Identified Novel Association for BMP4 with Non-Syndromic CL/P in an Asian Population Using Data from an International Cleft Consortium
Source: PLoS One. 2014 Oct 10;9(10):e109038. doi: 10.1371/journal.pone.0109038 (PMC4193821; doi:10.1371/journal.pone.0109038)
Supplement: Table S4 — Combined maternal exposure to environmental tobacco smoke, multivitamin supplements, alcohol consumption and tobacco smoking in NSCL/P probands from 681 complete European trios. (DOC) [file pone.0109038.s004.doc]

| Table S4 Combined maternal exposure to environmental tobacco smoke, multivitamin supplements, alcohol consumption and tobacco smoking in NSCL/P probands from 681 European trios | | | | | | |
| --- | --- | --- | --- | --- | --- | --- |
|  |  |  |  |  |  |  |
|  |  |  |  |  |  |  |
| Exposure | Alcohol | SMK | ETS | | | Total |
|  |  |  | Yes | No | *NA |  |
| VIT Yes | Yes | Yes | 4 | 19 | 26 | 49 |
|  |  | No | 6 | 36 | 41 | 83 |
|  |  | NA | 0 | 0 | 0 | 0 |
|  | No | Yes | 3 | 22 | 17 | 42 |
|  |  | No | 13 | 98 | 85 | 196 |
|  |  | NA | 0 | 0 | 0 | 0 |
|  | NA | Yes | 0 | 0 | 0 | 0 |
|  |  | No | 0 | 0 | 0 | 0 |
|  |  | NA | 0 | 0 | 0 | 0 |
| VIT No | Yes | Yes | 2 | 30 | 6 | 38 |
|  |  | No | 7 | 37 | 12 | 56 |
|  |  | NA | 0 | 0 | 0 | 0 |
|  | No | Yes | 3 | 19 | 10 | 32 |
|  |  | No | 11 | 64 | 18 | 93 |
|  |  | NA | 0 | 0 | 0 | 0 |
|  | NA | Yes | 0 | 0 | 0 | 0 |
|  |  | No | 0 | 0 | 0 | 0 |
|  |  | NA | 0 | 0 | 0 | 0 |
| VIT NA | Yes | Yes | 0 | 25 | 0 | 25 |
|  |  | No | 11 | 9 | 0 | 20 |
|  |  | NA | 0 | 0 | 0 | 0 |
|  | No | Yes | 0 | 20 | 0 | 20 |
|  |  | No | 4 | 17 | 3 | 24 |
|  |  | NA | 0 | 0 | 0 | 0 |
|  | NA | Yes | 0 | 0 | 0 | 0 |
|  |  | No | 0 | 0 | 1 | 1 |
|  |  | NA | 0 | 0 | 2 | 2 |
| Total | | | 64 | 396 | 221 | 681 |
| *NA: information missing | | | | | | |
