# Supplementary material for: Joint Testing of Genotypic and Gene-Environment Interaction Identified Novel Association for BMP4 with Non-Syndromic CL/P in an Asian Population Using Data from an International Cleft Consortium
Source: PLoS One. 2014 Oct 10;9(10):e109038. doi: 10.1371/journal.pone.0109038 (PMC4193821; doi:10.1371/journal.pone.0109038)
Supplement: Table S5 — Nominally significant associations for NSCL/P with SNPs in and around BMP4 jointly considering G and interaction with maternal VIT using conditional logistic regression models in 854 complete Asian trios informative for VIT. (DOC) [file pone.0109038.s005.doc]

| Table S5 Nominally significant associations for NSCL/P with SNPs in and around *BMP4* jointly considering G and interaction with maternal VIT using conditional logistic regression models in 854 complete Asian trios informative for VIT | | | | | | | | | | |
| --- | --- | --- | --- | --- | --- | --- | --- | --- | --- | --- |
|  |  |  |  |  |  |  |  |  |  |  |
| SNP name | Position | All Trios informative for VIT | | |  | Trios without exposure to VIT | | | | |
|  |  | MAF  (%) | *OR* (95%CI) _GxE | *P_*2df LRT(G+GxVIT) |  | MAF  (%) | | *OR* (95%CI) | | *P* |
| *rs7156227* | 54055337 | 20.8 | 1.19 (0.76, 1.86) | 4.20*10^-2^ |  | 20.6 | | 0.79 (0.66, 0.95) | | 1.28*10^-2^ |
| *rs1380131* | 54072858 | 9.5 | 2.03 (1.08, 3.84) | 5.01*10^-3^ |  | 9.2 | | 0.66 (0.51, 0.86) | | 2.23*10^-3^ |
| *rs210311* | 54123751 | 23.2 | 0.84 (0.54, 1.31) | 1.41*10^-1^ |  | 23.0 | | 1.19 (1.00, 1.42) | | 4.81*10^-2^ |
| *rs7154592* | 54143026 | 27.9 | 1.22 (0.82, 1.83) | 7.87*10^-2^ |  | 28.0 | | 0.83 (0.71, 0.98) | | 2.46*10^-2^ |
| *rs7146962* | 54145317 | 26.3 | 1.24 (0.71, 1.87) | 1.16*10^-1^ |  | 26.3 | | 0.84 (0.82, 1.87) | | 3.88*10^-2^ |
| *rs17127035* | 54358137 | 43.5 | 0.61 (0.42, 0.90) | 3.66*10^-2^ |  | 43.6 | | 1.10 (0.95, 1.28) | | 1.88*10^-1^ |
| *rs12435627* | 54367600 | 43.1 | 0.64 (0.44, 0.94) | 6.86*10^-2^ |  | 43.1 | | 1.05 (0.91, 1.22) | | 5.24*10^-1^ |
| *rs10498464* | 54371582 | 43.1 | 0.67 (0.45, 0.98) | 1.07*10^-1^ |  | 43.0 | | 1.04 (0.90, 1.21) | | 5.97*10^-1^ |
| *rs1951865* | 54372841 | 35.5 | 1.72 (1.15, 2.58) | 2.90*10^-2^ |  | 35.3 | | 0.91 (0.78, 1.06) | | 2.06*10^-1^ |
| *rs12879252* | 54375228 | 35.3 | 1.70 (1.14, 2.54) | 3.14*10^-2^ |  | 35.2 | | 0.90 (0.77, 1.05) | | 1.66*10^-1^ |
| *rs11157990* | 54383945 | 22.7 | 1.33 (0.83, 2.12) | 6.04*10^-2^ |  | 22.7 | | 0.81 (0.68, 0.97) | | 1.93*10^-2^ |
| *rs10498466* | 54391813 | 48.1 | 0.82 (0.57, 1.19) | 4.02*10^-3^ |  | 48.4 | | 1.29 (1.11, 1.49) | | 9.97*10^-4^ |
| *rs1957860* | 54429355 | 11.5 | 1.47 (0.84, 2.60) | 2.93*10^-2^ |  | 11.3 | | 0.73 (0.57, 0.92) | | 8.60*10^-3^ |
| *rs8014363* | 54431575 | 11.2 | 1.59 (0.86, 2.94) | 4.52*10^-2^ |  | 11.0 | | 0.75 (0.59, 0.95) | | 1.60*10^-2^ |
| *rs10873077* | 54433533 | 36.6 | 0.66 (0.45, 0.97) | 3.16*10^-2^ |  | 36.8 | | 1.19 (1.02, 1.39) | | 2.42*10^-2^ |
| *rs12878931* | 54549506 | 3.5 | 4.78 (1.25,18.18) | 3.74*10^-2^ |  | 3.7 | | 0.77 (0.52, 1.14) | | 1.93*10^-1^ |
| SNP name | Position | Trios had exposure to VIT | | |  | All trios informative for VIT(gTDT) | | | | |
|  |  | MAF(%) | *OR* (95%CI) | *P* |  | MAF(%) | *OR* (95%CI) | | *P* | |
| *rs7156227* | 54055337 | 21.8 | 0.94 (0.62, 1.41) | 7.56*10^-1^ |  | 20.8 | 0.81 (0.69, 0.96) | | 1.65*10^-2^ | |
| *rs1380131* | 54072858 | 11.0 | 1.35 (0.76, 2.41) | 3.09*10^-1^ |  | 9.5 | 0.75 (0.59, 0.95) | | 1.72*10^-2^ | |
| *rs210311* | 54123751 | 24.3 | 1.00 (0.67, 1.51) | 1.00 |  | 23.2 | 1.16 (0.99, 1.36) | | 6.85*10^-2^ | |
| *rs7154592* | 54143026 | 27.1 | 1.02 (0.70, 1.47) | 9.25*10^-1^ |  | 27.9 | 0.86 (0.74, 1.00) | | 4.29*10^-2^ | |
| *rs7146962* | 54145317 | 25.8 | 1.04 (0.71, 1.52) | 8.46*10^-1^ |  | 26.3 | 0.87 (0.75, 1.01) | | 6.90*10^-2^ | |
| *rs17127035* | 54358137 | 42.7 | 0.68 (0.48, 0.96) | 2.87*10^-2^ |  | 43.5 | 1.02 (0.89, 1.17) | | 7.29*10^-1^ | |
| *rs12435627* | 54367600 | 43.3 | 0.67 (0.47, 0.96) | 2.75*10^-2^ |  | 43.1 | 0.98 (0.86, 1.12) | | 7.82*10^-1^ | |
| *rs10498464* | 54371582 | 43.4 | 0.69 (0.49, 0.99) | 4.24*10^-2^ |  | 43.1 | 0.98 (0.85, 1.12) | | 7.55*10^-1^ | |
| *rs1951865* | 54372841 | 36.3 | 1.56 (1.07, 2.26) | 2.08*10^-2^ |  | 35.5 | 0.98 (0.85, 1.13) | | 7.99*10^-1^ | |
| *rs12879252* | 54375228 | 35.9 | 1.52 (1.05, 2.21) | 2.70*10^-2^ |  | 35.3 | 0.97 (0.84, 1.12) | | 6.89*10^-1^ | |
| *rs11157990* | 54383945 | 22.5 | 1.08 (0.70, 1.65) | 7.42*10^-1^ |  | 22.7 | 0.84 (0.72, 0.99) | | 4.13*10^-2^ | |
| *rs10498466* | 54391813 | 46.5 | 1.06 (0.76, 1.48) | 7.34*10^-1^ |  | 48.1 | 1.25 (1.09, 1.43) | | 1.65*10^-3^ | |
| *rs1957860* | 54429355 | 12.9 | 1.07 (0.64, 1.79) | 7.93*10^-1^ |  | 11.5 | 0.78 (0.63, 0.97) | | 2.23*10^-2^ | |
| *rs8014363* | 54431575 | 12.2 | 1.18 (0.67, 2.09) | 5.64*10^-1^ |  | 11.2 | 0.80 (0.64, 1.00) | | 4.48*10^-2^ | |
| *rs10873077* | 54433533 | 35.7 | 0.79 (0.55, 1.12) | 1.81*10^-1^ |  | 36.6 | 1.12 (0.97, 1.28) | | 1.25*10^-1^ | |
| *rs12878931* | 54549506 | 2.7 | 3.67 (1.02,13.14) | 4.61*10^-2^ |  | 3.5 | 0.92 (0.63, 1.32) | | 6.38*10^-1^ | |
