# Supplementary material for: Joint Testing of Genotypic and Gene-Environment Interaction Identified Novel Association for BMP4 with Non-Syndromic CL/P in an Asian Population Using Data from an International Cleft Consortium
Source: PLoS One. 2014 Oct 10;9(10):e109038. doi: 10.1371/journal.pone.0109038 (PMC4193821; doi:10.1371/journal.pone.0109038)
Supplement: Table S6 — Nominally significant associations for NSCL/P with SNPs in and around BMP4 jointly considering G and interaction with maternal ETS using conditional logistic regression models in 460 complete European trios informative for ETS. (DOC) [file pone.0109038.s006.doc]

| Table S6 Nominally significant associations for NSCL/P with SNPs in and around *BMP4* jointly considering G and interaction with maternal ETS using conditional logistic regression models in 460 complete European trios informative for ETS | | | | | | | | | | |
| --- | --- | --- | --- | --- | --- | --- | --- | --- | --- | --- |
|  |  |  |  |  |  |  |  |  |  |  |
|  |  |  |  |  |  |  |  |  |  |  |
| SNP name | Position | All Trios informative for ETS | | |  | Trios without exposure to ETS | | | | |
|  |  | MAF  (%) | *OR* (95%CI) _GxE | *P_*2df LRT  (G+GxETS) |  | MAF  (%) | *OR* (95%CI) | *P* | |  |
| *rs7156227* | 54055337 | 33.4 | 1.27 (0.70, 2.29) | 4.33*10^-1^ |  | 33.2 | 1.08 (0.87,1.34) | 5.09*10^-1^ | |  |
| *rs210327* | 54068781 | 43.3 | 0.46 (0.26, 0.81) | 2.16*10^-2^ |  | 43.1 | 1.11 (0.91,1.35) | 2.93*10^-1^ | |  |
| *rs1958654* | 54228517 | 14.6 | 0.51 (0.24, 1.06) | 4.69*10^-2^ |  | 14.6 | 1.39 (1.04,1.85) | 2.55*10^-2^ | |  |
| *rs8014363* | 54431575 | 46.4 | 0.56 (0.32, 0.97) | 9.50*10^-2^ |  | 46.7 | 1.03 (0.84,1.25) | 8.02*10^-1^ | |  |
| SNP name | Position | Trios had exposure to ETS | | |  | All trios informative for ETS(gTDT) | | |  | |
|  |  | MAF  (%) | *OR* (95%CI) | *P* |  | MAF  (%) | *OR* (95%CI) | *P* | |  |
| *rs7156227* | 54055337 | 34.3 | 1.36 (0.79, 2.36) | 2.69*10^-1^ |  | 33.4 | 1.11 (0.91, 1.36) | 3.06*10^-1^ | |  |
| *rs210327* | 54068781 | 44.5 | 0.51 (0.30, 0.87) | 1.27*10^-2^ |  | 43.3 | 1.00 (0.84, 1.21) | 9.63*10^-1^ | |  |
| *rs1958654* | 54228517 | 14.8 | 0.70 (0.35, 1.39) | 3.06*10^-1^ |  | 14.6 | 1.25 (0.96, 1.63) | 9.63*10^-2^ | |  |
| *rs8014363* | 54431575 | 44.5 | 0.58 (0.34, 0.96) | 3.45*10^-2^ |  | 46.4 | 0.95 (0.79, 1.14) | 5.77*10^-1^ | |  |
