# Supplementary material for: Joint Testing of Genotypic and Gene-Environment Interaction Identified Novel Association for BMP4 with Non-Syndromic CL/P in an Asian Population Using Data from an International Cleft Consortium
Source: PLoS One. 2014 Oct 10;9(10):e109038. doi: 10.1371/journal.pone.0109038 (PMC4193821; doi:10.1371/journal.pone.0109038)
Supplement: Table S7 — Nominally significant associations for NSCL/P with SNPs in and around BMP4 jointly considering G and interaction with maternal VIT using conditional logistic regression models in 589 complete European trios informative for VIT. (DOC) [file pone.0109038.s007.doc]

| Table S7 Nominally significant associations for NSCL/P with SNPs in and around *BMP4* jointly considering G and interaction with maternal VIT using conditional logistic regression models in 589 complete European trios informative for VIT | | | | | | | | |
| --- | --- | --- | --- | --- | --- | --- | --- | --- |
|  |  |  |  |  |  |  |  |  |
|  |  |  |  |  |  |  |  |  |
| SNP name | Position | All Trios informative for VIT | | |  | Trios without exposure to VIT | | |
|  |  | MAF(%) | *OR* (95%CI) _GxE | *P_*2dfLRT  (G+GxVIT) |  | MAF(%) | *OR* (95%CI) | *P* |
| *rs7156227* | 54055337 | 33.5 | 0.65 (0.45, 0.94) | 6.42*10^-2^ |  | 32.5 | 1.38 (1.02, 1.87) | 3.46*10^-2^ |
| *rs1380131* | 54072858 | 10.7 | 0.54 (0.30, 0.95) | 3.60*10^-2^ |  | 9.7 | 1.85 (1.15, 2.98) | 1.18*10^-2^ |
| *rs17126761* | 54148442 | 5.0 | 0.32 (0.15, 0.69) | 9.63*10^-2^ |  | 5.3 | 1.72 (0.96, 3.08) | 6.66*10^-2^ |
| *rs210360* | 54164706 | 32.5 | 0.75 (0.51, 1.08) | 1.42*10^-1^ |  | 30.5 | 1.35 (1.00, 1.83) | 4.96*10^-2^ |
| *rs210359* | 54167472 | 6.0 | 2.36 (1.11, 5.00) | 1.92*10^-2^ |  | 5.9 | 0.42 (0.23, 0.79) | 7.18*10^-3^ |
| *rs11157980* | 54310597 | 18.0 | 0.90 (0.58, 1.39) | 3.35*10^-2^ |  | 18.6 | 1.40 (1.00, 1.97) | 5.05*10^-2^ |
| SNP name | Position | Trios had exposure to VIT | | |  | All trios informative for VIT(gTDT) | | |
|  |  | MAF(%) | *OR* (95%CI) | *P* |  | MAF(%) | *OR* (95%CI) | *P* |
| *rs7156227* | 54055337 | 34.0 | 0.90 (0.73, 1.11) | 3.26*10^-1^ |  | 33.5 | 1.04 (0.87, 1.24) | 6.58*10^-1^ |
| *rs1380131* | 54072858 | 11.3 | 0.99 (0.72, 1.35) | 9.36*10^-1^ |  | 10.7 | 1.20 (0.93, 1.56) | 1.68*10^-1^ |
| *rs17126761* | 54148442 | 4.8 | 0.56 (0.34, 0.91) | 1.85*10^-2^ |  | 5.0 | 0.89 (0.62, 1.27) | 5.21*10^-1^ |
| *rs210360* | 54164706 | 33.7 | 1.01 (0.81, 1.25) | 9.55*10^-1^ |  | 32.5 | 1.12 (0.94, 1.33) | 2.23*10^-1^ |
| *rs210359* | 54167472 | 6.0 | 1.00 (0.66, 1.52) | 1.00 |  | 6.0 | 0.75 (0.54, 1.06) | 1.03*10^-1^ |
| *rs11157980* | 54310597 | 17.7 | 1.26 (0.97, 1.65) | 8.89*10^-2^ |  | 18.0 | 1.32 (1.07, 1.63) | 1.08*10^-2^ |
|  |  |  |  |  |  |  |  |  |
