# Supplementary material for: Joint Testing of Genotypic and Gene-Environment Interaction Identified Novel Association for BMP4 with Non-Syndromic CL/P in an Asian Population Using Data from an International Cleft Consortium
Source: PLoS One. 2014 Oct 10;9(10):e109038. doi: 10.1371/journal.pone.0109038 (PMC4193821; doi:10.1371/journal.pone.0109038)
Supplement: Table S8 — Significant and marginally significant associations for NSCL/P with SNPs in and around BMP4 jointly considering G and interaction with maternal ALCOHOL using conditional logistic regression models in 678 complete European trios informative for ALCOHOL. (DOC) [file pone.0109038.s008.doc]

| Table S8 Significant and marginally significant associations for NSCL/P with SNPs in and around *BMP4* jointly considering G and interaction with maternal ALCOHOL using conditional logistic regression models in 678 complete European trios informative for ALCOHOL | | | | | | | | |
| --- | --- | --- | --- | --- | --- | --- | --- | --- |
|  |  |  |  |  |  |  |  |  |
|  |  |  |  |  |  |  |  |  |
| SNP name | Position | All Trios informative for ALCOHOL | | |  | Trios without exposure to ALCOHOL | | |
|  |  | MAF  (%) | *OR* (95%CI) _GxE | *P_*2df LRT  (G+GxALCOHOL) |  | MAF(%) | *OR* (95%CI) | *P* |
| *rs7156227* | 54055337 | 33.9 | 1.44 (1.04, 2.01) | 7.66*10^-2^ |  | 33.9 | 0.91 (0.74, 1.12) | 3.64*10^-1^ |
| *rs210388* | 54099700 | 31.4 | 0.68 (0.49, 0.94) | 6.75*10^-2^ |  | 31.6 | 1.14 (0.92, 1.40) | 2.26*10^-1^ |
| *rs8020341* | 54101869 | 8.3 | 0.43 (0.24, 0.79) | 1.35*10^-2^ |  | 9.4 | 1.13 (0.82, 1.57) | 4.55*10^-1^ |
| *rs210386* | 54102009 | 32.6 | 0.69 (0.50, 0.96) | 5.13*10^-2^ |  | 33.2 | 1.07 (0.87, 1.30) | 5.38*10^-1^ |
| *rs210313* | 54123352 | 23.0 | 0.61 (0.42, 0.90) | 4.13*10^-2^ |  | 23.4 | 1.21 (0.96, 1.53) | 1.01*10^-1^ |
| *rs210311* | 54123751 | 22.5 | 0.60 (0.41, 0.88) | 3.16*10^-2^ |  | 22.9 | 1.21 (0.96, 1.52) | 1.13*10^-1^ |
| *rs210302* | 54153996 | 23.5 | 0.62 (0.43. 0.91) | 4.80*10^-2^ |  | 23.8 | 1.20 (0.96, 1.51) | 1.15*10^-1^ |
| *rs210370* | 54157966 | 28.3 | 0.64 (0.45, 0.91) | 4.33*10^-2^ |  | 28.3 | 1.17 (0.94, 1.45) | 1.54*10^-1^ |
| *rs11157980* | 54310597 | 18.0 | 0.88 (0.58, 1.31) | 4.21*10^-2^ |  | 19.2 | 1.34 (1.05, 1.72) | 2.10*10^-2^ |
| SNP name | Position | Trios had exposure to ALCOHOL | | |  | All trios informative for ALCOHOL(gTDT) | | |
|  |  | MAF  (%) | *OR* (95%CI) | *P* |  | MAF(%) | *OR* (95%CI) | *P* |
| *rs7156227* | 54055337 | 31.2 | 1.31 (1.01, 1.69) | 3.86*10^-2^ |  | 33.9 | 1.05 (0.90, 1.24) | 5.37*10^-1^ |
| *rs210388* | 54099700 | 27.2 | 0.77 (0.59, 1.00) | 4.86*10^-2^ |  | 31.4 | 0.98 (0.83, 1.15) | 7.73*10^-1^ |
| *rs8020341* | 54101869 | 4.7 | 0.49 (0.29, 0.81) | 5.94*10^-3^ |  | 8.3 | 0.88 (0.70, 1.15) | 3.37*10^-1^ |
| *rs210386* | 54102009 | 28.1 | 0.73 (0.57, 0.95) | 1.91*10^-2^ |  | 32.6 | 0.93 (0.79, 1.08) | 3.33*10^-1^ |
| *rs210313* | 54123352 | 19.5 | 0.75 (0.55, 1.01) | 5.68*10^-2^ |  | 23.0 | 1.01 (0.84, 1.22) | 8.89*10^-1^ |
| *rs210311* | 54123751 | 19.1 | 0.72 (0.53, 0.98) | 3.75*10^-2^ |  | 22.5 | 1.00 (0.83, 1.20) | 1.00 |
| *rs210302* | 54153996 | 20.1 | 0.75 (0.56, 1.01) | 5.97*10^-2^ |  | 23.5 | 1.01 (0.84, 1.21) | 9.26*10^-1^ |
| *rs210370* | 54157966 | 25.0 | 0.75 (0.56, 0.99) | 4.05*10^-2^ |  | 28.3 | 0.99 (0.83, 1.17) | 8.96*10^-1^ |
| *rs11157980* | 54310597 | 13.8 | 1.17 (0.85, 1.61) | 3.31*10^-1^ |  | 18.0 | 1.27 (1.05, 1.55) | 1.54*10^-2^ |
