# Supplementary material for: Joint Testing of Genotypic and Gene-Environment Interaction Identified Novel Association for BMP4 with Non-Syndromic CL/P in an Asian Population Using Data from an International Cleft Consortium
Source: PLoS One. 2014 Oct 10;9(10):e109038. doi: 10.1371/journal.pone.0109038 (PMC4193821; doi:10.1371/journal.pone.0109038)
Supplement: Table S9 — Significant and marginally significant associations for NSCL/P with SNPs in and around BMP4 jointly considering G and interaction with maternal SMK using conditional logistic regression models in 679 complete European trios informative for SMK. (DOC) [file pone.0109038.s009.doc]

| Table S9 Significant and marginally significant associations for NSCL/P with SNPs in and around *BMP4* jointly considering G and interaction with maternal SMK using conditional logistic regression models in 679 complete European trios informative for SMK | | | | | | | | |
| --- | --- | --- | --- | --- | --- | --- | --- | --- |
|  |  |  |  |  |  |  |  |  |
|  |  |  |  |  |  |  |  |  |
| SNP name | Position | All Trios informative for SMK | | |  | Trios without exposure to SMK | | |
|  |  | MAF  (%) | *OR* (95%CI) _GxE | *P_*2df LRT  (G+GxSMK) |  | MAF  (%) | *OR* (95%CI) | *P* |
| rs1999345 | 54041955 | 47.2 | 0.96 (0.69, 1.32) | 8.81*10^-2^ |  | 47.5 | 1.20 (1.00, 1.43) | 4.80*10^-2^ |
| rs7156227 | 54055337 | 33.9 | 1.08 (0.76, 1.52) | 7.79*10^-1^ |  | 33.5 | 1.03 (0.84, 1.25) | 8.04*10^-1^ |
| rs2150276 | 54311027 | 34.2 | 1.28 (0.90, 1.82) | 7.94*10^-2^ |  | 34.7 | 1.08 (0.89, 1.30) | 4.42*10^-1^ |
| rs7148896 | 54316607 | 30.2 | 1.40 (0.97, 2.02) | 5.67*10^-2^ |  | 30.8 | 1.04 (0.86, 1.26) | 6.96*10^-1^ |
| SNP name | Position | Trios had exposure to SMK | | |  | All trios informative for SMK(gTDT) | | |
|  |  | MAF  (%) | *OR* (95%CI) | *P* |  | MAF  (%) | *OR* (95%CI) | *P* |
| rs1999345 | 54041955 | 46.7 | 1.14 (0.87, 1.50) | 3.34*10^-1^ |  | 47.2 | 1.18 (1.02, 1.37) | 2.90*10^-2^ |
| rs7156227 | 54055337 | 34.7 | 1.10 (0.83, 1.47) | 5.08*10^-1^ |  | 33.9 | 1.05 (0.89, 1.23) | 5.64*10^-1^ |
| rs2150276 | 54311027 | 33.2 | 1.38 (1.02, 1.86) | 3.56*10^-2^ |  | 34.2 | 1.16 (0.99, 1.36) | 7.46*10^-2^ |
| rs7148896 | 54316607 | 28.7 | 1.46 (1.06, 1.99) | 1.91*10^-2^ |  | 30.2 | 1.14 (0.97, 1.34) | 1.15*10^-1^ |
