# Supplementary material for: Joint Testing of Genotypic and Gene-Environment Interaction Identified Novel Association for BMP4 with Non-Syndromic CL/P in an Asian Population Using Data from an International Cleft Consortium
Source: PLoS One. 2014 Oct 10;9(10):e109038. doi: 10.1371/journal.pone.0109038 (PMC4193821; doi:10.1371/journal.pone.0109038)
Supplement: Table S10 — Nominally significant associations with NSCL/P for SNPs in and around BMP4 jointly considering G and interactions with maternal ETS and VIT using conditional logistic regression models in 746 complete Asian trios informative for ETS and VIT. (DOC) [file pone.0109038.s010.doc]

| Table S10 Nominally significant associations with NSCL/P for SNPs in and around *BMP4* jointly considering G and interactions with maternal ETS and VIT using conditional logistic regression models in 746 complete Asian trios informative for ETS and VIT | | | | | | | | |
| --- | --- | --- | --- | --- | --- | --- | --- | --- |
|  |  |  |  |  |  |  |  |  |
|  |  |  |  |  |  |  |  |  |
|  |  |  |  |  |  |  |  |  |
| *SNPs* | Position |  | GxETS | |  | GxVIT | | *P_*3df LRT |
|  |  |  | *OR* (95%CI) | *P* |  | *OR* (95%CI) | *P* |  |
| *rs7152946* | *54051076* |  | 1.80 (1.14, 2.82) | 1.11*10^-2^ |  | 0.95 (0.48, 1.89) | 8.91*10-1 | 4.20*10^-2^ |
| *rs7156227* | *54055337* |  | 1.64 (1.13, 2.37) | 9.46*10^-3^ |  | 1.18 (0.71, 1.97) | 5.26*10^-1^ | 4.13*10^-4^ |
| *rs1380131* | 54072858 |  | 1.01 (0.58, 1.75) | 9.67*10^-1^ |  | 2.20 (1.05, 4.58) | 3.58*10^-2^ | 1.50*10^-3^ |
| *rs10483623* | *54090077* |  | 0.71 (0.39, 1.28) | 2.51*10^-1^ |  | 0.57 (0.26, 1.27) | 1.69*10^-1^ | 1.17*10^-1^ |
| *rs210313* | *54123352* |  | 0.88 (0.62, 1.25) | 4.66*10^-1^ |  | 0.82 (0.49, 1.36) | 4.37*10^-1^ | 1.48*10^-1^ |
| *rs210311* | *54123751* |  | 0.88 (0.62, 1.25) | 4.76*10^-1^ |  | 0.77 (0.46, 1.29) | 3.20*10^-1^ | 7.74*10^-2^ |
| *rs7154592* | *54143026* |  | 1.13 (0.82, 1.57) | 4.56*10^-1^ |  | 1.21 (0.77, 1.90) | 4.08*10^-1^ | 8.33*10^-2^ |
| *rs7146962* | *54145317* |  | 1.19 (0.85, 1.67) | 3.09*10^-1^ |  | 1.24 (0.77, 1.98) | 3.75*10^-1^ | 1.01*10^-1^ |
| *rs17127035* | *54358137* |  | 1.19 (0.88, 1.61) | 2.68*10^-1^ |  | 0.58 (0.37, 0.90) | 1.44*10^-2^ | 4.23*10^-2^ |
| *rs12435627* | *54367600* |  | 1.10 (0.81, 1.48) | 5.55*10^-1^ |  | 0.58 (0.37, 0.91) | 1.66*10^-2^ | 7.95*10^-2^ |
| *rs2181733* | *54368872* |  | 1.12 (0.83, 1.52) | 4.54*10^-1^ |  | 0.63 (0.41, 0.98) | 4.16*10^-2^ | 1.49*10^-1^ |
| *rs10498464* | *54371582* |  | 1.16 (0.86, 1.57) | 3.42*10^-1^ |  | 0.63 (0.41, 0.99) | 4.26*10^-2^ | 1.26*10^-1^ |
| *rs1951865* | *54372841* |  | 0.82 (0.60, 1.12) | 2.07*10^-1^ |  | 2.10 (1.30, 3.39) | 2.32*10^-3^ | 5.11*10^-3^ |
| *rs12879252* | *54375228* |  | 0.83 (0.61, 1.14) | 2.58*10^-1^ |  | 2.13 (1.32, 3.43) | 1.93*10^-3^ | 5.51*10^-3^ |
| *rs11157990* | *54383945* |  | 1.30 (0.91, 1.85) | 1.48*10^-1^ |  | 1.46 (0.87, 2.46) | 1.56*10^-1^ | 1.17*10^-1^ |
| *rs10498466* | *54391813* |  | 0.83 (0.61, 1.12) | 2.23*10^-1^ |  | 0.76 (0.50, 1.15) | 1.94*10^-1^ | 2.40*10^-2^ |
| *rs1957860* | *54429355* |  | 1.46 (0.91, 2.35) | 1.17*10^-1^ |  | 1.68 (0.88, 3.21) | 1.19*10^-1^ | 5.47*10^-2^ |
| *rs8014363* | *54431575* |  | 1.55 (0.96, 2.51) | 7.65*10^-2^ |  | 1.72 (0.84, 3.51) | 1.36*10^-1^ | 6.56*10^-2^ |
| *rs10873077* | *54433533* |  | 0.91 (0.66, 1.26) | 5.79*10^-1^ |  | 0.63 (0.40, 0.99) | 4.32*10^-2^ | 1.21*10^-1^ |
| *rs12878931* | 54549506 |  | 1.69 (0.72, 3.96) | 2.30*10^-1^ |  | 5.98 (1.20,29.71) | 2.89*10^-2^ | 7.25*10^-2^ |
| *SNPs* | Position | Trios without exposure to either ETS or VIT | | |  | All trios informative for ETS&VIT(gTDT) | | |
|  |  | MAF  (%) | *OR* (95%CI) | *P* |  | *OR* (95%CI) | *P* | MAF(%) |
| *rs7152946* | *54051076* | 11.0 | 0.68 (0.49, 0.93) | 1.53*10^-2^ |  | 0.87 (0.70, 1.09) | 2.16*10^-1^ | 11.9 |
| *rs7156227* | *54055337* | 20.1 | 0.58 (0.45, 0.76) | 4.47*10^-5^ |  | 0.74 (0.61, 0.88) | 8.71*10^-4^ | 20.8 |
| *rs1380131* | 54072858 | 10.2 | 0.58 (0.41, 0.81) | 1.65*10^-3^ |  | 0.65 (0.50, 0.84) | 1.06*10^-3^ | 9.4 |
| *rs10483623* | *54090077* | 6.4 | 1.63 (1.08, 2.46) | 2.06*10^-2^ |  | 1.29 (0.98, 1.72) | 7.42*10^-2^ | 6.9 |
| *rs210313* | *54123352* | 23.7 | 1.30 (1.02, 1.65) | 3.15*10^-2^ |  | 1.20 (1.01, 1.42) | 3.73*10^-2^ | 23.6 |
| *rs210311* | *54123751* | 22.8 | 1.34 (1.05, 1.70) | 1.70*10^-2^ |  | 1.23 (1.03, 1.46) | 1.92*10^-2^ | 23.0 |
| *rs7154592* | *54143026* | 27.5 | 0.76 (0.61, 0.95) | 1.71*10^-2^ |  | 0.83 (0.70, 0.97) | 1.88*10^-2^ | 27.5 |
| *rs7146962* | *54145317* | 25.4 | 0.75 (0.60, 0.95) | 1.67*10^-2^ |  | 0.84 (0.71, 0.99) | 3.24*10^-2^ | 25.8 |
| *rs17127035* | *54358137* | 45.5 | 1.03 (0.84, 1.26) | 7.91*10^-1^ |  | 1.02 (0.88, 1.18) | 7.94*10^-1^ | 43.8 |
| *rs12435627* | *54367600* | 45.0 | 1.01 (0.83, 1.23) | 9.16*10^-1^ |  | 0.98 (0.84, 1.13) | 7.38*10^-1^ | 43.5 |
| *rs2181733* | *54368872* | 45.0 | 0.99 (0.81, 1.21) | 9.07*10^-1^ |  | 0.97 (0.84, 1.13) | 7.10*10^-1^ | 43.5 |
| *rs10498464* | *54371582* | 45.2 | 0.98 (0.80, 1.20) | 9.34*10^-1^ |  | 0.98 (0.84, 1.13) | 7.36*10^-1^ | 43.5 |
| *rs1951865* | *54372841* | 34.9 | 1.01 (0.82, 1.25) | 9.06*10^-1^ |  | 1.03 (0.88, 1.20) | 7.27*10^-1^ | 36.4 |
| *rs12879252* | *54375228* | 34.7 | 1.00 (0.81, 1.23) | 9.80*10^-1^ |  | 1.02 (0.88, 1.19) | 7.86*10^-1^ | 36.2 |
| *rs11157990* | *54383945* | 23.1 | 0.75 (0.59, 0.96) | 2.15*10^-2^ |  | 0.88 (0.74, 1.05) | 1.58*10^-1^ | 23.3 |
| *rs10498466* | *54391813* | 48.7 | 1.35 (1.11, 1.66) | 3.06*10^-3^ |  | 1.21 (1.05, 1.40) | 1.05*10^-2^ | 48.0 |
| *rs1957860* | *54429355* | 11.9 | 0.65 (0.48, 0.89) | 7.80*10^-3^ |  | 0.81 (0.65, 1.02) | 7.45*10^-2^ | 11.8 |
| *rs8014363* | *54431575* | 11.7 | 0.65 (0.47, 0.91) | 1.03*10^-2^ |  | 0.83 (0.66, 1.05) | 1.25*10^-1^ | 11.4 |
| *rs10873077* | *54433533* | 37.2 | 1.21 (0.99, 1.49) | 6.50*10^-2^ |  | 1.10 (0.95, 1.28) | 2.04*10^-1^ | 35.7 |
| *rs12878931* | 54549506 | 4.1 | 0.69 (0.41, 1.17) | 1.68*10^-1^ |  | 0.98 (0.66, 1.45) | 9.21*10^-1^ | 3.7 |
