# Supplementary material for: Joint Testing of Genotypic and Gene-Environment Interaction Identified Novel Association for BMP4 with Non-Syndromic CL/P in an Asian Population Using Data from an International Cleft Consortium
Source: PLoS One. 2014 Oct 10;9(10):e109038. doi: 10.1371/journal.pone.0109038 (PMC4193821; doi:10.1371/journal.pone.0109038)
Supplement: Table S11 — Nominally significant associations for NSCL/P with SNPs in and around BMP4 jointly considering G and interactions with maternal SMK, ETS, ALCOHOL and VIT using conditional logistic regression models in 374 complete European trios informative for all four exposures. (DOC) [file pone.0109038.s011.doc]

| Table S11 Nominally significant associations for NSCL/P with SNPs in and around *BMP4* jointly considering G and interactions with maternal SMK, ETS, ALCOHOL and VIT using conditional logistic regression models in 374 complete European trios informative for all four exposures | | | | | | | | | | |  |
| --- | --- | --- | --- | --- | --- | --- | --- | --- | --- | --- | --- |
|  |  |  |  |  |  |  |  |  |  |  |  |
|  |  |  |  |  |  |  |  |  |  |  |  |
|  |  |  |  |  |  |  |  |  |  |  |  |
| *SNPs* | Position | GxSMK | |  | GxETS | |  | GxVIT | | MAF(%)  (no exposure on any one of the four factors) | |
|  |  | *OR* (95%CI) | *P* |  | *OR* (95%CI) | *P* |  | *OR* (95%CI) | *P* |  |  |
| *rs210327* | 54068781 | 0.73 (0.46, 1.17) | 1.89*10^-1^ |  | 0.38 (0.20, 0.71) | 2.63*10^-3^ |  | 1.11 (0.74, 1.68) | 6.10*10^-1^ | 42.4 | |
| *rs1380131* | 54072858 | 1.38 (0.63, 2.99) | 4.19*10^-1^ |  | 5.55 (1.50,20.49) | 1.02*10^-2^ |  | 0.50 (0.24, 1.03) | 6.16*10^-2^ | 10.4 | |
| *rs210361* | 54164036 | 1.36 (0.77, 2.42) | 2.92*10^-1^ |  | 1.44 (0.68, 3.03) | 3.38*10^-1^ |  | 1.27 (0.77, 2.09) | 3.41*10^-1^ | 24.0 | |
| *rs7156227* | [54055337](http://www.ncbi.nlm.nih.gov/sites/nuccore/NC_000014.8?report=graph&v=54054837:54055837&content=5&m=54055337!&mn=rs7156227&dispmax=1&currpage=1) | 0.92 (0.56, 1.51) | 7.39*10^-1^ |  | 1.06 (0.52, 2.17) | 8.65*10^-1^ |  | 0.64 (0.41, 1.02) | 6.26*10^-2^ | 28.9 | |
| *SNPs* | Position | GxALCOHOL | |  | Trios without exposure to any of the four exposures | |  | All trios informative for all four exposures (gTDT) | | *P*_5df LRT | |
|  |  | *OR* (95%CI) | *P* |  | *OR* (95%CI) | *P* |  | *OR* (95%CI) | P |  |  |
| *rs210327* | 54068781 | 1.15 (0.75, 1.78) | 5.27*10^-1^ |  | 1.15 (0.81, 1.63) | 4.45*10^-1^ |  | 1.03 (0.84, 1.26) | 7.58*10^-1^ | 4.99*10^-2^ | |
| *rs1380131* | 54072858 | 0.83 (0.40, 1.72) | 6.11*10^-1^ |  | 1.55 (0.76, 3.17) | 2.29*10^-1^ |  | 1.25 (0.90, 1.75) | 1.80*10^-1^ | 1.90*10^-2^ | |
| *rs210361* | 54164036 | 1.51 (0.88, 2.58) | 1.37*10^-1^ |  | 0.64 (0.41, 0.99) | 4.55*10^-2^ |  | 0.96 (0.76, 1.22) | 7.61*10^-1^ | 2.12*10^-1^ | |
| *rs7156227* | [54055337](http://www.ncbi.nlm.nih.gov/sites/nuccore/NC_000014.8?report=graph&v=54054837:54055837&content=5&m=54055337!&mn=rs7156227&dispmax=1&currpage=1) | 1.28 (0.78, 2.09) | 3.32*10^-1^ |  | 1.34 (0.86, 2.06) | 1.93*10^-1^ |  | 1.11 (0.89, 1.39) | 3.61*10^-1^ | 2.94*10^-1^ | |
